# AKAP13 couples GPCR signaling to mTORC1 inhibition

**DOI:** 10.1371/journal.pgen.1009832

**Published:** 2021-10-21

**Authors:** Shihai Zhang, Huanyu Wang, Chase H. Melick, Mi-Hyeon Jeong, Adna Curukovic, Shweta Tiwary, Tshering D. Lama-Sherpa, Delong Meng, Kelly A. Servage, Nicholas G. James, Jenna L. Jewell

**Affiliations:** 1 Department of Molecular Biology, University of Texas Southwestern Medical Center, Dallas, Texas, United States of America; 2 Harold C. Simmons Comprehensive Cancer Center, University of Texas Southwestern Medical Center, Dallas, Texas, United States of America; 3 Hamon Center for Regenerative Science and Medicine, University of Texas Southwestern Medical Center, Dallas, Texas, United States of America; 4 Provincial Key Laboratory of Animal Nutrition Control, College of Animal Science, South China Agricultural University, Guangzhou, People’s Republic of China; 5 Howard Hughes Medical Institute, University of Texas Southwestern Medical Center, Dallas, Texas, United States of America; 6 Department of Cell and Molecular Biology, John A. Burns School of Medicine, University of Hawaii, Honolulu, Hawaii, United States of America; Rutgers Robert Wood Johnson Medical School, UNITED STATES

## Abstract

The mammalian target of rapamycin complex 1 (mTORC1) senses multiple stimuli to regulate anabolic and catabolic processes. mTORC1 is typically hyperactivated in multiple human diseases such as cancer and type 2 diabetes. Extensive research has focused on signaling pathways that can activate mTORC1 such as growth factors and amino acids. However, less is known about signaling cues that can directly inhibit mTORC1 activity. Here, we identify A-kinase anchoring protein 13 (AKAP13) as an mTORC1 binding protein, and a crucial regulator of mTORC1 inhibition by G-protein coupled receptor (GPCR) signaling. GPCRs paired to Gα_s_ proteins increase cyclic adenosine 3’5’ monophosphate (cAMP) to activate protein kinase A (PKA). Mechanistically, AKAP13 acts as a scaffold for PKA and mTORC1, where PKA inhibits mTORC1 through the phosphorylation of Raptor on Ser 791. Importantly, AKAP13 mediates mTORC1-induced cell proliferation, cell size, and colony formation. AKAP13 expression correlates with mTORC1 activation and overall lung adenocarcinoma patient survival, as well as lung cancer tumor growth *in vivo*. Our study identifies AKAP13 as an important player in mTORC1 inhibition by GPCRs, and targeting this pathway may be beneficial for human diseases with hyperactivated mTORC1.

## Author summary

The mammalian target of rapamycin complex 1 (mTORC1) can sense multiple upstream stimuli to regulate cell growth and metabolism. Increased mTORC1 activation results in many human diseases such as cancer. Small molecules like rapamycin that target and inhibit mTORC1, are available in the clinic with limited success. Thus, decoding the mechanisms involved in mTORC1 regulation is crucial. Most of the research has focused on stimuli that activate mTORC1. Less is known about signaling pathways that can directly inhibit mTORC1 activity. G-protein coupled receptors (GPCRs) coupled to Gα_s_ proteins signal to and potently inhibit mTORC1. In this study, we have identified AKAP13 to play a crucial role in mTORC1 inhibition by GPCR signaling. Importantly, GPCRs are the largest family of drug targets with many approved FDA compounds. Targeting this signaling pathway may be beneficial for human diseases with hyperactivated mTORC1.

## Introduction

The mammalian target of rapamycin (mTOR) is an evolutionarily conserved Ser/Thr kinase, that is often referred to as the “master regulator” of cell growth because it promotes anabolic events and inhibits catabolic processes [1–3]. mTOR is the catalytic component of a complex referred to as mTOR complex 1 (mTORC1), which includes regulatory-associated protein of mTOR (Raptor) and mammalian lethal with Sec13 protein 8 (mLST8, also known as GβL). Raptor recognizes mTORC1 substrates and mLST8 acts a positive regulator of mTOR activity. Hyperactivated mTORC1 is observed in many diseases including cancer, type 2 diabetes, and neurodegeneration. Currently, rapamycin and analogs (referred to as rapalogs) are used clinically to inhibit mTORC1 activity. However, these drugs have been shown to have multiple limitations. For example, rapamycin is cytostatic instead of cytotoxic, fails to inhibit some downstream mTORC1-mediated processes, and several feedback loops activate upon mTORC1 inhibition [4]. Thus, understanding the molecular mechanisms involved in mTORC1 regulation is important for the development of new therapeutic approaches.

Upstream stimuli such as growth factors, amino acids, energy status, and stress modulate mTORC1 activity [1–3]. Elevated intracellular amino acid concentrations promote mTORC1 lysosomal localization and subsequent activation through a Rag GTPase-dependent [5] or Rag GTPase-independent mechanism [6,7]. Once mTORC1 is at the lysosome, it comes into close contact with and is activated by the small G-protein, Ras homolog enriched in brain (Rheb). The last 15 amino acids of Rheb, including the CAAX (C = Cys, A = aliphatic, X = terminal amino acid) box, facilitate its lysosomal localization [8,9]. Guanosine triphosphate (GTP)-bound Rheb directly interacts with mTORC1, resulting in a conformational change in the mTOR active site and subsequent allosteric activation of the kinase [10]. Growth factors signal through Rheb to activate mTORC1 by stimulating the dissociation of the tuberous sclerosis complex (TSC) away from the lysosome and Rheb [8]. TSC is a GTPase activating protein (GAP) for Rheb, resulting in Rheb bound to guanosine diphosphate (GDP) and inactivating it [11–13]. TSC mutations constitutively activate mTORC1 and result in the human diseases TSC or lymphangioleiomyomatosis (LAM) [14–16]. Thus, amino acids and growth factor signaling converge at the lysosome to achieve optimal mTORC1 activation.

GPCRs paired to Gα_s_ proteins potently inhibit the activity of mTORC1 in numerous cell lines [17]. Activation of GPCR-Gα_s_ signaling elevates cyclic adenosine 3’5’ monophosphate (cAMP) levels, resulting in the activation of the well characterized Ser/Thr kinase Protein Kinase A (PKA). The PKA holoenzyme is comprised of two catalytic subunits and two regulatory subunits [18]. Upon cAMP binding to the PKA regulatory subunits, the regulatory subunits will undergo a conformational change, releasing the catalytic subunits and resulting in the active form of PKA [19]. PKA regulates many physiological events [20] and its cellular localization is largely dependent on the A kinase anchoring proteins (AKAPs), which anchor PKA to distinct compartments and provide specificity in signaling cascades [21,22]. So far, over 70 AKAPs have been identified that participate in a variety of cellular processes [23,24]. Importantly, GPCRs are the largest group of cell surface receptors comprising more than 1% of the human genome, and are the main family of drug targets with many FDA approved compounds [25,26].

To further understand the molecular mechanisms by which GPCRs can inhibit mTORC1, we investigated the role of AKAP13 in this signaling pathway. AKAP13 acts as a scaffold for PKA and mTORC1, which results in Raptor Ser 791 phosphorylation and mTORC1 inhibition. Importantly, AKAP13 plays a key role in mTORC1-mediated biology and the overall survival of lung cancer patients.

## Results

### AKAP13 interacts with and regulates mTORC1 activity

**Fig 1:**
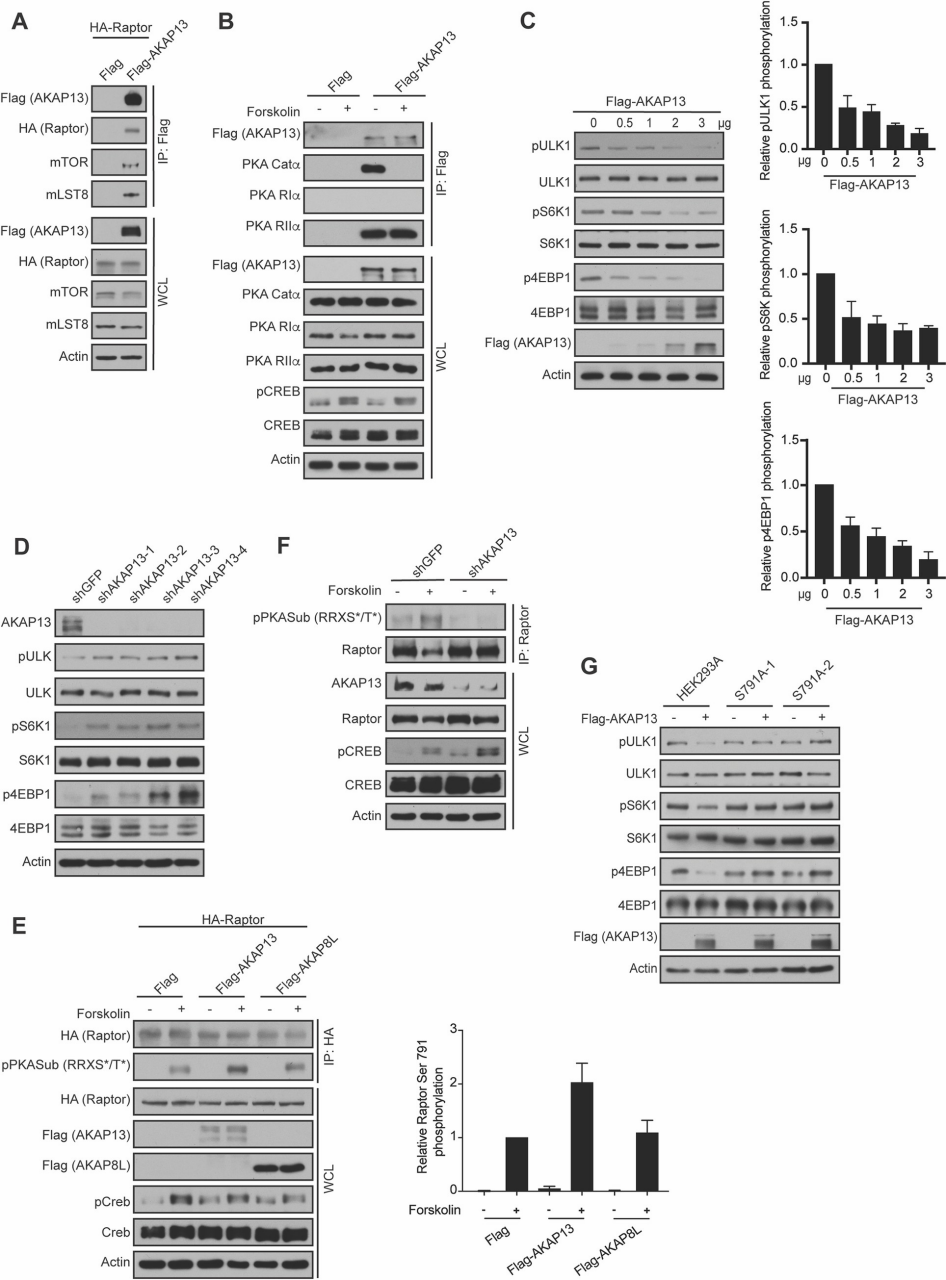
AKAP13 binds to and regulates mTORC1 activity. **(A)** AKAP13 interacts with mTORC1. Empty vector (Flag) or Flag-tagged AKAP13 was co-expressed with HA-tagged Raptor in HEK293A cells. 24 hours later Flag immunoprecipitates (IPs) were analyzed by immunoblotting for Flag-tagged AKAP13, HA-tagged Raptor, mTOR, and mLST8. Actin was used as a loading control. WCL denotes whole cell lysate. **(B)** AKAP13 interacts with PKA Catα and PKA RIIα. Empty vector (Flag) or Flag-tagged AKAP13 was co-expressed with HA-tagged Raptor in HEK293A cells. 24 hours later cells were then treated with or without 10 μM forskolin and 200 μM IBMX for 1 h, and Flag immunoprecipitates (IPs) were analyzed by immunoblotting for Flag-tagged AKAP13, PKA Catα, PKA RIα, and PKA RIIα. CREB phosphorylation (pCREB) at Ser 133 was used as a positive control for forskolin stimulation. Actin and CREB were used as loading controls. WCL denotes whole cell lysate. **(C)** *Left-* Elevated AKAP13 levels decrease mTORC1 activity. Flag-tagged AKAP13 (0–3 μg) was overexpressed in HEK293A cells for 24 hours. mTORC1 activity was analyzed by protein immunoblotting for the phosphorylation status of S6K1 (pS6K1) at Thr 389, 4EBP1 (p4EBP1) at Thr 37 and Thr 46, and ULK1 (pULK1) at Ser 758. S6K, 4EBP1, ULK1, and Actin were probed as loading controls. *Right*- Quantification of phospho-ULK1. P-Values: 0 μg FLAG-tagged AKAP13 vs 0.5 μg FLAG-tagged AKAP13 p<0.05, 0 μg FLAG-tagged AKAP13 vs 1 μg FLAG-tagged AKAP13 p<0.01, 0 μg FLAG-tagged AKAP13 vs 2 μg FLAG-tagged AKAP13 p<0.0001 and 0 μg FLAG-tagged AKAP13 vs 3 μg FLAG-tagged AKAP13 p<0.001. Quantification of phospho-S6K. P-values: 0 μg FLAG-tagged AKAP13 vs 0.5 μg FLAG-tagged AKAP13 p = 0.05, 0 μg FLAG-tagged AKAP13 vs 1 μg FLAG-tagged AKAP13 p<0.01, 0 μg FLAG-tagged AKAP13 vs 2 μg FLAG-tagged AKAP13 p< 0.01 and 0 μg FLAG-tagged AKAP13 vs 3 μg FLAG-tagged AKAP13 p<0.0001. Quantification of phospho-4EBP1. P-values: 0 μg FLAG-tagged AKAP13 vs 0.5 μg FLAG-tagged AKAP13 p<0.05, 0 μg FLAG-tagged AKAP13 vs 1 μg FLAG-tagged AKAP13 p<0.01, 0 μg FLAG-tagged AKAP13 vs 2 μg FLAG-tagged AKAP13 p<0.001 and 0 μg FLAG-tagged AKAP13 vs 3 μg FLAG-tagged AKAP13 p<0.001. **(D)** Decreased AKAP13 levels increase mTORC1 activity. HEK293A stable cell lines expressing control shRNA (shGFP) or four different shRNA targeting AKAP13 (shAKAP13 1–4) were generated. mTORC1 activity and loading controls were analyzed as described in (C). **(E)** *Left-* Elevated AKAP13 levels increase Raptor Ser 791 phosphorylation. HA-tagged Raptor was co-expressed with empty vector (Flag), Flag-tagged AKAP13, or Flag-tagged AKAP8L in HEK293A cells. 24 hours later cells were then treated with or without 10 μM forskolin and 200 μM IBMX for 1 hour, and HA immunoprecipitates (IPs) were analyzed by immunoblotting for HA-tagged Raptor, and phospho-PKA substrate antibody (pPKASub (RRXS*/T*)). CREB phosphorylation (pCREB) at Ser 133 was used as a positive control for forskolin stimulation. Actin and CREB were used as loading controls. WCL denotes whole cell lysate. *Right-* Quantification of phospho-PKA substrate (pPKASub (RRXS*/T*) to determine Raptor Ser 791 phosphorylation. P-values: Flag -Forskolin vs Flag +Forskolin p<0.0001, Flag-tagged AKAP13 -Forskolin vs Flag-tagged AKAP13 +Forskolin p<0.01, Flag-tagged AKAP8L -Forskolin vs Flag-tagged AKAP8L +Forskolin p<0.01, Flag +Forskolin vs Flag-tagged AKAP8L -Forskolin p<0.0001, Flag +Forskolin vs Flag-tagged AKAP13 +Forskolin p<0.05. **(F)** Decreased AKAP13 levels decrease Raptor Ser 791 phosphorylation. HEK293A stable cell lines expressing control shRNA (shGFP) or shRNA targeting AKAP13 (shAKAP13) were generated. HEK2943A cells were then treated with or without 10 μM forskolin and 200 μM IBMX for 1 hour, and Raptor immunoprecipitates (IPs) were analyzed by immunoblotting for Raptor and phospho-PKA substrate antibody (pPKASub (RRXS*/T*)). CREB phosphorylation (pCREB) at Ser 133 was used as a positive control for forskolin stimulation. Actin and CREB were used as loading controls. WCL denotes whole cell lysate. **(G)** Elevated AKAP13 inhibits mTORC1 though Raptor Ser 791 phosphorylation. Flag-tagged AKAP13 was overexpressed for 24 hours in HEK293A or HEK293A Raptor Ser 791 mutant cells (S791A-1, S791A-2). mTORC1 activity and loading controls were analyzed as described in (C).

To identify potential AKAPs involved in GPCR signaling to mTORC1, we immunopurified the mTORC1 complex via Flag-tagged Raptor. Mass spectrometry analysis of anti-Flag immunoprecipitants identified AKAP13 (also known as AKAP-Lbc), as well as the PKA regulatory subunit IIα (PKA RIIα). We confirmed that both AKAP13 and PKA RIIα subunits are mTORC1 interacting proteins by co-immunoprecipitation experiments. Flag-tagged AKAP13 interacted with HA-tagged Raptor and mTORC1 components (mTOR and mLST8) in HEK293A cells (**Fig 1A**). A previous study showed that AKAP13 contains a PKA RIIα subunit binding site [27]. Consistently, we found that Flag-tagged AKAP13 binds to the PKA RIIα subunit and the PKA catalytic α subunit (PKA Catα), but not the PKA regulatory subunit Iα in HEK293A cells (**Fig 1B**). As expected, cells treated with forskolin, a pharmacological activator of adenyl cyclase, resulted in the dissociation of the PKA Catα subunit from the AKAP13-PKA RIIα complex. Thus, AKAP13 forms a complex with mTORC1, PKA RIIα subunit, and PKA Catα subunit.

To determine whether AKAP13 regulates mTORC1 activity, we overexpressed Flag-tagged AKAP13 and an unrelated AKAP called AKAP8L (**Figs 1C and S1**) and analyzed mTORC1 activity in HEK293A cells by the phosphorylation of its substrates (S6K at Thr 389, 4EBP1 at Thr 37 and Thr 46, and ULK1 at Ser 758). Phosphorylation of S6K and 4EBP1 promote protein synthesis, whereas phosphorylation of ULK1 inhibits autophagy [28,29]. Although overexpression of Flag-tagged AKAP8L did not alter mTORC1 activity (**S1 Fig**), the expression of Flag-tagged AKAP13 inhibited mTORC1 activity in a dose-dependent manner (**Fig 1C**). Consistently, knockdown of AKAP13 using four different short hairpin RNAs (shRNA, shAKAP13 1–4) enhanced the activity of mTORC1 (**Fig 1D**). Recently, we identified that GPCRs paired to Gα_s_ proteins inhibit the activity of mTORC1 via Raptor Ser 791 phosphorylation [17]. Thus, we investigated whether AKAP13 altered mTORC1 activity through Raptor Ser 791 phosphorylation. HA-tagged Raptor was co-expressed with either vector control (Flag), Flag-tagged AKAP8L, or Flag-tagged AKAP13 in HEK293A cells that were treated with or without forskolin. HA-tagged Raptor was immunoprecipitated, and the phosphorylation of Raptor on Ser 791 was analyzed using a phospho-PKA substrate antibody (pPKA Sub (R-R-X-S*/T*)). This antibody specifically recognizes Raptor Ser 791 phosphorylation, when Raptor is immunoprecipitated [17]. Interestingly, co-expression of HA-tagged Raptor with Flag-tagged AKAP13 significantly increased Raptor Ser 791 phosphorylation (**Fig 1E**), consistent with overexpression of Flag-tagged AKAP13 inhibiting mTORC1 (**Fig 1C**). In contrast, co-expression of HA-tagged Raptor with Flag-tagged AKAP8L did not significantly change Raptor Ser 791 phosphorylation. Moreover, knockdown of AKAP13 (shAKAP13) in HEK293A cells completely abolished phosphorylation of Raptor on Ser 791 **(Fig 1F)**. To further confirm AKAP13 inhibits mTORC1 activity through the phosphorylation of Raptor at Ser 791, we utilized HEK293A cell lines bearing the endogenous Raptor Ser 791 to Ala 791 (S791A) mutation. The homozygous Raptor S791A knock-ins (clones S791A-1 and S791A-2) were generated and verified as previously described [17]. Flag-tagged AKAP13 overexpression decreased mTORC1 activity in wild type HEK293A cells, but not in HEK293A S791A-1 and S791A-2 cells (**Fig 1G)**. Therefore, AKAP13 regulates the activity of mTORC1 through Raptor Ser 791 phosphorylation.

### AKAP13 scaffolds mTORC1 next to the PKA holoenzyme

**Fig 2:**
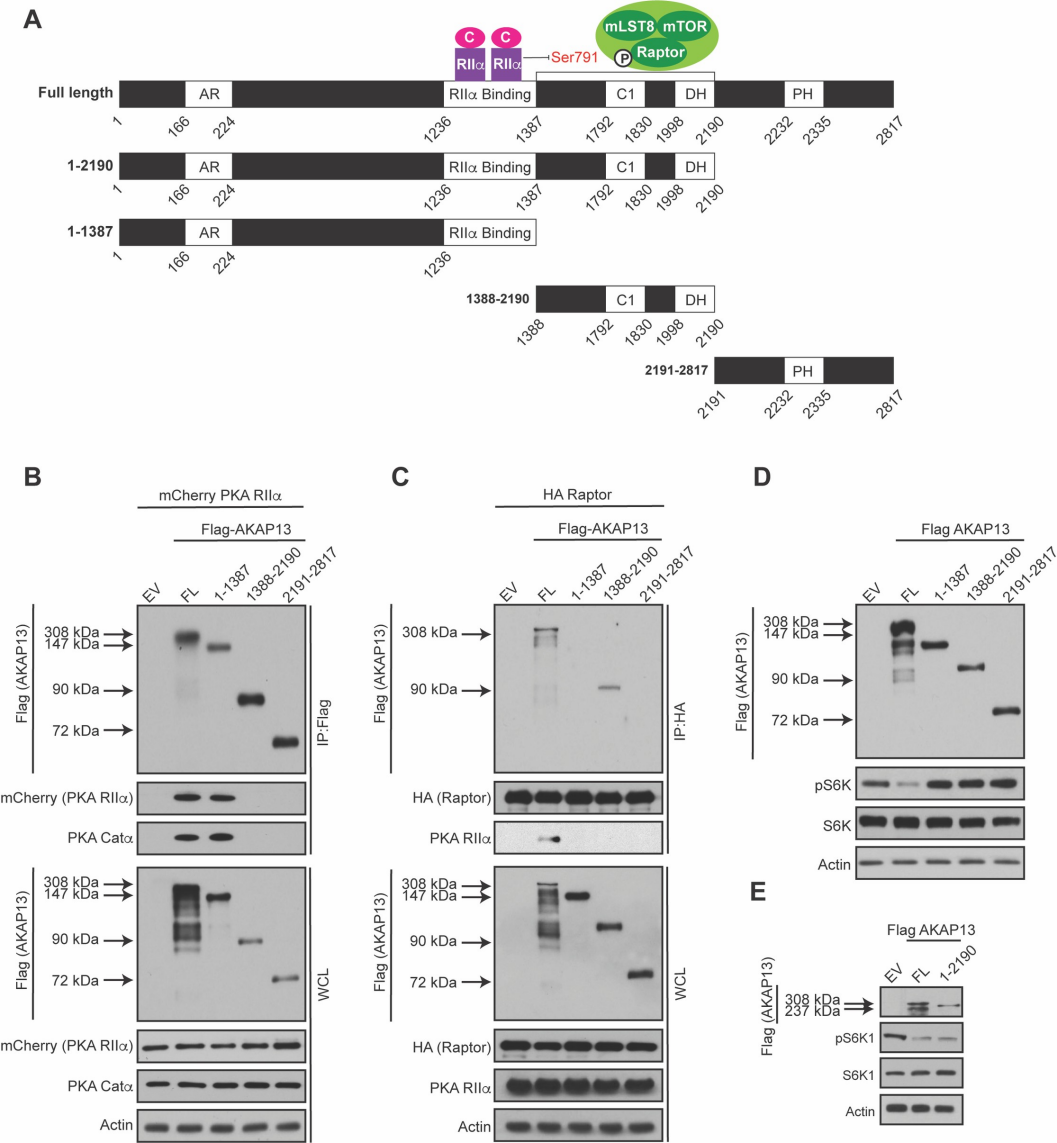
AKAP13 scaffolds PKA and mTORC1 in close proximity. **(A)** Schematic of the different Flag-tagged AKAP13 truncations generated for experiments. In addition, an illustration of how the PKA holoenzyme (PKA Catα and PKA RIIα subunits) and mTORC1 bind to AKAP13 according to the results shown below. **(B)** PKA Catα and PKA RIIα subunits interact with AKAP13. mCherry-tagged PKA RIIα was co-expressed with either empty vector (EV), Flag-tagged AKAP13 full-length (FL), or different Flag-tagged AKAP13 truncations (1–1387, 1388–2190, 2191–2817). 24 hours later Flag immunoprecipitates (IPs) were analyzed by immunoblotting for Flag-tagged AKAP13 FL or truncations, mCherry-tagged PKA RIIα, and PKA Catα. Actin was used as a loading control. WCL denotes whole cell lysate. **(*C*)** Raptor interacts with AKAP13 between amino acids 1388 and 2190. HA-tagged Raptor was co-expressed with either empty vector (EV), Flag-tagged AKAP13 full-length (FL), or different Flag-tagged AKAP13 truncations (1–1387, 1388–2190, 2191–2817). 24 hours later HA immunoprecipitates (IPs) were analyzed by immunoblotting for Flag-tagged AKAP13 FL or truncations, HA-tagged Raptor, or PKA RIIα. Actin was used as a loading control. WCL denotes whole cell lysate. **(*D*)** Elevated full-length AKAP13 levels decrease mTORC1 activity. Empty vector (EV), Flag-tagged AKAP13 full-length (FL), or different Flag-tagged AKAP13 truncations (1–1387, 1388–2190, 2191–2817) were overexpressed in HEK293A cells for 24 hours. mTORC1 activity was analyzed by protein immunoblotting for the phosphorylation status of S6K1 (pS6K1). S6K and Actin were probed as loading controls. **(*E*)** The region of AKAP13 that contains the PKA holoenzyme and mTORC1 regulates mTORC1 activity. Empty vector (EV), Flag-tagged AKAP13 full-length (FL), or Flag-tagged AKAP13 amino acids 1–2190 (1–2190) were overexpressed in HEK293A cells for twenty-four hours. mTORC1 activity was analyzed by protein immunoblotting for the phosphorylation status of S6K1 (pS6K1). S6K and Actin were probed as loading controls.

AKAP13 serves as a scaffold for multiple protein kinases including PKA, Protein Kinase C (PKC), and Protein Kinase D (PKD) [30]. AKAP13 is a 2817 amino acid protein containing multiple domains [31] (**Fig 2A**). For example, AKAP13 contains ankyrin repeats (AR) involved in protein-protein interactions, a PKA RIIα subunit domain (RIIα Binding) that anchors PKA to AKAP13, a C1 domain that binds to phorbol esters and stimulates protein kinase activity, a dbl homology (DH) domain that is associated with the guanine exchange factor (GEF) activity for the small GTPase RhoA, and a pleckstrin homology (PH) domain. Previous work showed that AKAP13 is a guanine exchange factor (GEF) for RhoA promoting p38α activation [32]. Because it was reported that p38α promotes TSC-14-3-3 binding and TSC regulates mTORC1 activity [33, 34], we tested if the GEF activity of AKAP13 regulates mTORC1. Depletion of p38α using small interfering RNA (siRNA) did not alter the activity of mTORC1 (**S2A Fig**). Moreover, AKAP13 was shown to enhance the ERK signaling cascade [35], and ERK has been reported to inhibit TSC to promote mTORC1 activation [36]. To determine whether the ERK pathway altered Raptor Ser 791 phosphorylation, HEK293A cells were treated with or without ERK inhibitors (SCH77 and PD03) and Raptor Ser 791 phosphorylation was analyzed (**S2B Fig**). As expected, cells treated with forskolin increased Raptor Ser 791 phosphorylation, and cells treated with ERK inhibitors decreased the phosphorylation of ERK [17,37,38]. However, cells pretreated with ERK inhibitors did not alter Raptor Ser 791 phosphorylation. Thus, AKAP13 GEF activity and the role of AKAP13 in ERK signaling does not appear to regulate Raptor Ser 791 phosphorylation and mTORC1 activity.

In order to map where mTORC1 interacts with AKAP13, we designed four different AKAP13 truncations based on a previous study (**Fig 2A)** [31]. It was shown that the PKA RIIα binds to AKAP13 [27, 31]. To confirm the binding between AKAP13 and PKA RIIα, mCherry-tagged PKA RIIα was co-expressed with either full-length (FL) Flag-tagged AKAP13 (amino acids 1–2817), or Flag-tagged AKAP13 truncations (amino acids 1-1387, 1388–2190, or 2191–2817). Full-length Flag-tagged AKAP13 and Flag-tagged AKAP13 1–1387 (which contains the PKA RIIα binding region) were able to interact with mCherry-tagged PKA RIIα and PKA Catα (**Fig 2B**). In contrast, Flag-tagged AKAP13 1388–2190 and Flag-tagged AKAP13 2191–2817, which do not contain the PKA RIIα binding region, could not interact with mCherry-tagged PKA RIIα. Next to map where mTORC1 interacted with AKAP13, HA-tagged Raptor was co-expressed with either full length Flag-tagged AKAP13 (amino acids 1–2817) or different Flag-tagged AKAP13 truncations (amino acids 1–1387, 1388–2190, or 2191–2817). HA-tagged Raptor interacted with full-length Flag-tagged AKAP13 and Flag-tagged AKAP13 1388–2190 (**Fig 2C**). Thus, mTORC1 interacts with AKAP13 within amino acids 1388–2190, near the PKA holoenzyme.

Because elevated levels of Flag-tagged AKAP13 can inhibit mTORC1 (**Fig 1C**), we wanted to determine what region of AKAP13 is important for regulating the activity of mTORC1. Full-length Flag-tagged AKAP13 or the Flag-tagged AKAP13 truncations (**Fig 2A**) were overexpressed and mTORC1 activity was assessed (**Fig 2D and 2E**). AKAP13 truncations which lack the PKA RIIα binding region, mTORC1 binding region, or both, were not able to inhibit mTORC1 activity. Interestingly, overexpression of Flag-tagged AKAP13 truncation 1–2190 (which contains both the PKA RIIα and mTORC1 binding region) inhibits mTORC1 activity similar to the overexpression of full-length Flag-tagged AKAP13 (**Fig 2E**). Taken together, the region of AKAP13 that contains both the PKA holoenzyme and mTORC1 binding region is necessary for the regulation of mTORC1 activity.

### AKAP13 and mTORC1 interact in the absence of amino acids

**Fig 3:**
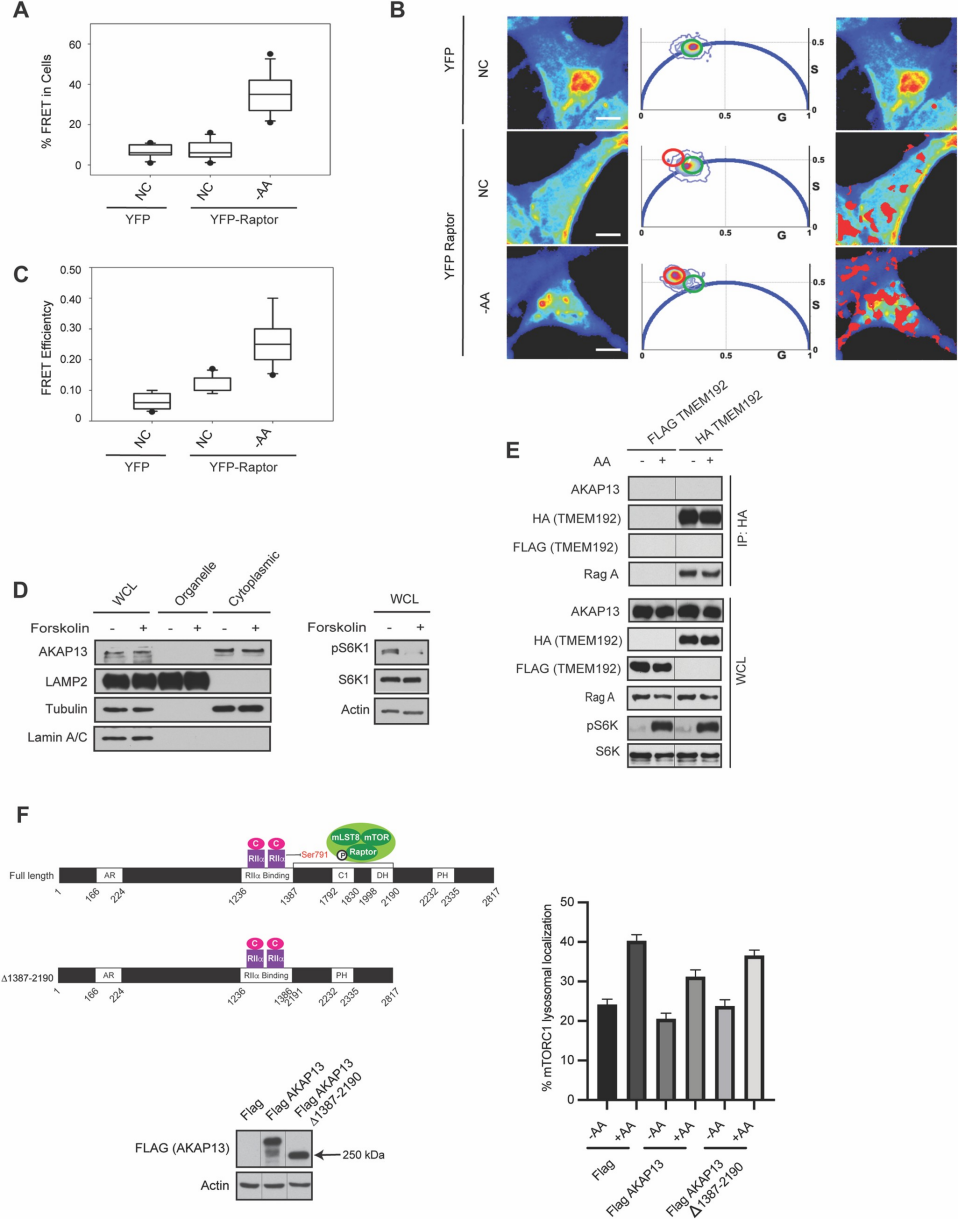
AKAP13 and mTORC1 interact under amino acid deficient conditions. **(*A*)** AKAP13-Raptor interaction is increased in the absence of amino acids in human embryonic kidney 293 (HEK293A) cells. The binding between GFP-tagged AKAP13 and YFP-tagged Raptor was assessed under the different conditions: 1) Normal conditions (NC) containing amino acids; and 2) Amino acid deficient conditions (-AA) where the cells were starved of amino acids for 1 h. There was significant increase in AKAP13-Raptor interaction in amino acid deficient conditions when compared to normal conditions. P-values: GFP-tagged AKAP13 + Control (YFP) vs GFP-tagged AKAP13 + YFP-tagged Raptor -AA p<0.0001, GFP-tagged AKAP13 + YFP-tagged Raptor NC vs GFP-tagged AKAP13 +YFP-tagged Raptor -AA p<0.0001. **(B)** The first panel shows intensity images of HEK293A cells with GFP-tagged AKAP13 co-transfected with YFP (FRET control) or YFP-tagged Raptor (first panel). The middle panel shows the phasor distribution of 1) YFP with no FRET (Green circle) and 2) YFP with high FRET (red circle). The third panel shows spatial distribution of pixels associated FRET highlighting the differences in FRET under different conditions. **(C)** Box plots showing FRET efficiency of HEK293A cells co-transfected with GFP-tagged AKAP13 and YFP-tagged Raptor under the different conditions described in (A). There was significant increase in AKAP13-Raptor FRET efficiency in amino acid deficient conditions when compared to normal conditions, indicating that AKAP13 and Raptor are closer in proximity under these conditions. P-values: GFP-tagged AKAP13 + Control (YFP) vs GFP-tagged AKAP13 + YFP-tagged Raptor NC p<0.001, GFP-tagged AKAP13 + Control (YFP) vs GFP-tagged AKAP13 + YFP-tagged Raptor -AA p<0.0001, GFP-tagged AKAP13 + YFP-tagged Raptor NC vs GFP-tagged AKAP13 + YFP-tagged Raptor -AA p<0.0001. **(*D*)** AKAP13 resides in the cytoplasm. *Left*—Subcellular fractionation experiments in HEK293A cells were performed separating the organelle and cytoplasmic fraction. AKAP13, LAMP2 (lysosomal marker), Tubulin (cytoplasmic marker), and Lamin A/C (nuclear marker) were immunoblotted. *Right*–mTORC1 activity was analyzed by protein immunoblotting for the phosphorylation status of S6K1 (pS6K1) at Thr 389. Actin were probed as loading control. **(*E*)** AKAP13 does not localize to the lysosome. MIA Paca-2 cells stably expressing Flag-tagged or HA-tagged TMEM192 were starved of amino acids (-AA) for 2 hours, and then stimulated with amino acids (+AA) for 2 hours. HA-tagged TMEM192 was immunoprecipitated with HA beads and IP and WCL were probed for AKAP13, HA or RagA. Flag, pS6K and S6K were probed for as controls. **(*F*)** *Left*–HEK293A cells were transfected with Flag, wild-type Flag-tagged AKAP13, and Flag-tagged AKAP13 Δ1387–2190 constructs. *Right*–After 24 hours, cells were starved of amino acids for 2 hours, and then stimulated with amino acids for 1 hour. mTOR and LAMP2 (lysosomal marker) co-localization was quantified. P-values: Flag -AA vs Flag +AA p<0.0001, Flag +AA vs Flag-tagged AKAP13 +AA p<0.001, Flag-tagged AKAP13 -AA vs Flag-tagged AKAP13 +AA p<0.0001, Flag-tagged AKAP13 Δ1387–2190 -AA vs Flag-tagged AKAP13 Δ 1387–2190 +AA p<0.0001.

Under amino acid sufficient conditions mTORC1 is lysosomal localized where it becomes activated by Rheb [9]. When amino acids are depleted mTORC1 is not at the lysosome, and dispersed throughout the cell at an unknown location. We previously showed that increased intracellular cAMP levels can inhibit amino acid signaling to mTORC1 through both the Rag GTPase-dependent [5] and the Rag GTPase-independent pathway [6,7]. However, cAMP levels did not alter mTORC1 lysosomal localization, but still blocked amino acid-induced mTORC1 activation through an unknown mechanism. Because AKAP13 and mTORC1 bind in cells (**Fig 1A**), we investigated whether AKAP13 and mTORC1 interact in the absence or presence of amino acids. Fluorescence lifetime imaging microscopy (FLIM) combined with Förster resonance energy transfer (FRET; FLIM/FRET) (**Fig 3A, 3B and 3C**) were used to examine the interaction between AKAP13 with Raptor in live-cells under different conditions. Specifically, YFP-tagged Raptor and EGFP-tagged AKAP13 were overexpressed and observed for live cell studies in HEK293A cells. Under normal culturing conditions with sufficient amino acids, there was a low percentage (~7%) of FRET between YFP-tagged Raptor and EGFP-tagged AKAP13 (**Fig 3A and 3B**). In contrast, a significant increase in complex formation between AKAP13 and mTORC1 (~30%) was observed in cells that were starved of amino acids, suggesting that AKAP13 and mTORC1 do not form a complex at the lysosome. Similarly, there was low FRET efficiency between YFP-tagged Raptor and GFP-tagged AKAP13 (~0.11) in culturing conditions with amino acids, and a significant increase in FRET efficiency when starved of amino acids (~0.27) (**Fig 3C**). Subcellular fractionation experiments reveal that AKAP13 is found in the cytoplasm and not the fraction containing lysosomes (**Fig 3D**). In support of the FRET and subcellular fractionation data, lysosome immunoprecipitations (Lyso-IPs) were performed where we immunopurified lysosomes from cells expressing transmembrane protein 192 (TMEM192, lysosome localized protein) [39]. AKAP13 is not localized at the lysosome, like lysosomal associated proteins RagA in amino acid depleted cells or when cells have amino acids available (**Fig 3E**). Interestingly, overexpression of Flag-tagged AKAP13 can decrease mTORC1 lysosomal localization, whereas Flag-tagged AKAP13 missing the mTORC1 binding region (Flag-tagged AKAP13 Δ1387–2190) did not significantly decrease mTORC1 lysosomal localization (**Fig 3F**). Thus, AKAP13 and mTORC1 co-localize under amino acid depleted conditions away from the lysosome.

**Fig 4:**
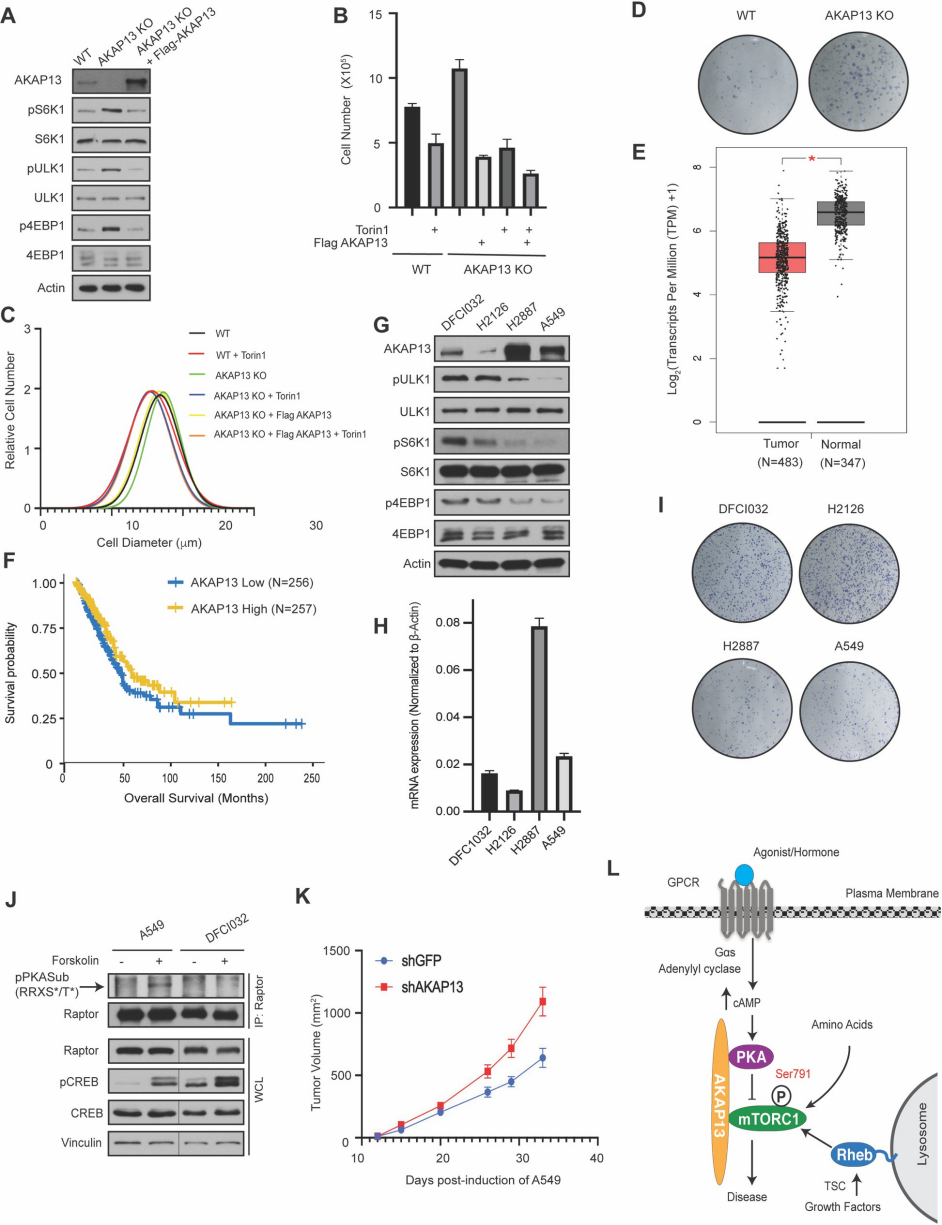
AKAP13 regulates mTORC1 mediated biology and lung cancer tumorigenesis. **(A)** The level of AKAP13 regulates mTORC1 activity. Wild-type (WT), AKAP13 knock-out (KO), or AKAP13 KO expressing Flag-tagged AKAP13 human embryonic kidney 293 (HEK293A) cells were assessed. mTORC1 activity was analyzed by protein immunoblotting for the phosphorylation status of S6K1 (pS6K1) at Thr 389, 4EBP1 (p4EBP1) at Thr 37 and Thr 46, and ULK1 (pULK1) at Ser 758. S6K, 4EBP1, ULK1, and Actin were probed as loading controls. (**B)** The level of AKAP13 regulates cell proliferation. Proliferation in wild-type (WT), AKAP13 knock-out (KO), or AKAP13 KO expressing Flag-tagged AKAP13 HEK293A cells with or without Torin1 was analyzed. P- values: WT -Torin1 vs WT +Torin1 p<0.05, WT -Torin1 vs AKAP13 KO -Torin1 p<0.05, WT -Torin1 vs AKAP13 KO + Flag-tagged AKAP13 -Torin1 p<0.001, AKAP13 KO -Torin1 vs AKAP13 KO +Torin1 p<0.01, AKAP13 KO + Flag-tagged AKAP13 -Torin1 vs AKAP13 KO + Flag-tagged AKAP13 +Torin1 p<0.01, WT +Torin1 vs AKAP13 KO +Flag-tagged AKAP13 +Torin1 p<0.05, AKAP13 KO -Torin1 vs AKAP13 KO + Flag-tagged AKAP13 -Torin1 p<0.001. **(C)** The level of AKAP13 regulates cell size. Cell size in wild-type (WT), AKAP13 knock-out (KO), or AKAP13 KO expressing Flag-tagged AKAP13 HEK293A cells treated with or without Torin1 was analyzed. P-values: WT -Torin1 vs WT +Torin1 p<0.001, WT -Torin1 vs AKAP13 KO -Torin1 p<0.01, AKAP13 KO -Torin1 vs AKAP13 KO +Torin1 p<0.0001, AKAP13 KO + Flag-tagged AKAP13 -Torin1 vs AKAP13 KO + Flag-tagged AKAP13 +Torin1 p<0.001, WT +Torin1 vs AKAP13 KO +Torin1 p<0.05, AKAP13 KO -Torin1 vs AKAP13 KO + Flag-tagged AKAP13 -Torin1 p<0.0001. **(D)** The level of AKAP13 regulates colony formation. Colony formation experiments in wild-type (WT) and AKAP13 knock-out (KO) HEK293A cells was analyzed. **(E)** AKAP13 levels are downregulated in lung adenocarcinoma (LUAD). Data was derived from GEPIA database (http://gepia.cancer-pku.cn). **(F)** AKAP13 levels correlate with LUAD overall survival. A TCGA cohort of LUAD was divided into two groups according to the mRNA expression levels of AKAP13. Overall survival was compared between these two groups, as shown in Kaplan-Meier curves. Numbers of patients in each group, log-rank P values, and hazard ratios (H) with 95% confidence interval (CI) are indicated. p = 0.037. **(G)** AKAP13 levels correlate with mTORC1 activity in LUAD cell lines. AKAP13 proteins levels and mTORC1 activity were assessed in LUAD cell lines (DFCI032, H2126, H2887 and A549). mTORC1 activity and controls were analyzed as in (*A*). **(H)** AKAP13 mRNA expression level parallels protein expression level in LUAD cell lines DFC1032, H2125, H2887, and A549. The quantitative mRNA expression (2^-ΔΔ*CT*^) in LUAD cells were determined by real-time quantitative PCR. P-values: DFC1032 cells vs. H2126 cells p<0.001, DFC1032 cells vs. H2887 cells p<0.0001, DFC1032 cells vs. A549 cells p<0.01, H2126 cells vs. H2887 cells p<0.0001, H2887 cells vs. A549 cells p<0.0001, H2126 cells vs. A549 p<0.0001. **(I)** AKAP13 levels correlate with colony formation in LUAD cell lines. Colony formation experiments in LUAD cell lines (DFCI032, H2126, H2887 and A549) was analyzed. **(J)** Cells with higher AKAP13 levels have an increase in Raptor Ser 791 phosphorylation. A549 or DFC1032 cells were treated with or without 10 μM forskolin and 200 μM IBMX, and Raptor was immunoprecipitated (IP) analyzed by immunoblotting with a PKA substrate antibody that recognizes RRXS*/T* (pPKA Sub (RRXS*/T*)). CREB phosphorylation was included as a positive control for forskolin stimulation. CREB, Raptor, and Vinculin are controls. WCL denotes whole cell lysate. **(K)** Decreased levels of AKAP13 increases tumor volume. Subcutaneous xenografts were performed on NOD SCID mice using stable A549 cell lines expressing control shRNA (shGFP) or shRNA targeting AKAP13 (shAKAP13). P-values: Day 15 shGFP vs shAKAP13 p<0.05, Day 26 shGFP vs shAKAP13 p<0.05, Day 29 shGFP vs shAKAP13 p<0.05, Day 33 shGFP vs shAKAP13 p<0.05. **(L)** Working model of how GPCR signaling inhibits mTORC1. Increased cAMP levels activate PKA to phosphorylate Raptor at Ser 791 resulting in mTORC1 inhibition. AKAP13 scaffolds mTORC1 next to the PKA holoenzyme in the cytoplasm.

### AKAP13 regulates mTORC1-mediated biology and lung cancer tumorigenesis

mTORC1 regulates a variety of physiological processes like protein translation, cell size, and cell proliferation [1–3]. To test if AKAP13 regulates mTORC1-mediated biology, AKAP13 was knocked out (KO) in HEK293A cells via the CRISPR-Cas9 system (**Fig 4A**). mTORC1 activity was significantly increased in AKAP13 KO cells, like what was seen when AKAP13 was depleted using shRNA (**Fig 1D**). AKAP13 KO cells significantly increased cell proliferation (**Fig 4B**) and cell size (**Fig 4C**). Likewise, reconstitution experiments with Flag-tagged AKAP13 in AKAP13 KO cells reduced cell size and proliferation (**Fig 4B and 4C**). Moreover, AKAP13 KO cells treated with Torin1 (a mTOR inhibitor) reduced cell size and proliferation. Cell colony formation was increased in AKAP13 KO cells when compared to wild type cells (**Fig 4D**). Therefore, AKAP13 appears to regulate several mTORC1-mediated processes, including cell size, cell proliferation, and colony formation.

Since AKAP13 regulates mTORC1-mediated biology, we wanted to explore the role of AKAP13 in tumorigenesis. According to the data from Gene Expression Profiling Interactive Analysis (GEPIA, http://gepia.cancer-pku.cn/), AKAP13 mRNA expression is downregulated in lung adenocarcinoma (LUAD) (**Fig 4E**). We found that high expression of AKAP13 increases Raptor Ser 791 phosphorylation and inhibits mTORC1 activity (**Fig 1C and 1E**). Consistently, higher AKAP13 mRNA expression positively correlates with a high clinical survival rate in LUAD (**Fig 4F**). Four different LUAD cancer cell lines (DFCI032, H2126, H2887 and A549) were utilized to analyze AKAP13 protein level and mTORC1 activity. RNA sequencing data [40], immunoblot, and mRNA analysis corresponding with DFCI032 and H2126 having low AKAP13 expression, whereas H2887 and A549 have high AKAP13 expression (**Fig 4G and 4H**). Importantly, cells with higher AKAP13 expression showed lower mTORC1 activity. Consistently, lung cancer cells (H2887, A549) with high AKAP13 expression formed less colonies than lung cancer cells (DFC1032, H2126) with low AKAP13 expression (**Fig 4I**). Consistently, cells with high AKAP13 levels had elevated Raptor Ser 791 phosphorylation (**Fig 4J**). Xenograft experiments were performed in mice that were injected subcutaneously with A549 cells (with high AKAP13 expression), with or without AKAP13 depletion (**Fig 4K**). Interestingly, in cells with AKAP13 depletion the tumor volume was significantly increased. Next, we tested if a phospho-mimetic of Raptor Ser 791 (HA-tagged Raptor Ser 791 to Asp 791, HA-tagged Raptor S791D) could affect cell proliferation and size in AKAP13 KO or LUAD cancer cells (**S3 Fig**). Overexpression of a Raptor Ser 791 phosphomimetic did not alter cell proliferation or size. However, it is well-known that phosphomimetics (Asp or Glu mutants) don’t always mimic the phosphorylation status of a protein. In addition to LUAD cells we also observed a decrease in mTORC1 activity (as measured by the phosphorylation of S6) when AKAP13 was overexpressed in prostate (PC3) and liver (Huh7) cancer cells (**S4 Fig**). Thus, AKAP13 expression level correlates mTORC1-mediated biology, tumorigenesis *in vivo*, and overall patient survival in LUAD.

## Discussion

mTORC1 senses multiple upstream stimuli to regulate anabolic and catabolic events within the cell [1–3]. GPCR-Gα_s_ signaling increases cAMP levels and regulates mTORC1 activity [17]. Elevated cAMP levels activate PKA to directly phosphorylate Raptor at Ser 791 resulting in mTORC1 inhibition. Here, we show that AKAP13 scaffolds PKA next to mTORC1 away from the lysosome, resulting in the phosphorylation of Raptor at Ser 791 and mTORC1 inhibition (**Fig 4L**). Modulating AKAP13 levels alters Raptor Ser 791 phosphorylation and the activation of mTORC1. Consistently, AKAP13 doesn’t affect Raptor Ser 791 phosphorylation or mTORC1 activity in Raptor S791A (Raptor Ser 791 phosphodeficient) knock-in HEK293A cell lines. AKAP13 also regulates mTORC1-mediated biology such as cell size, cell proliferation, and tumorigenesis *in vivo*. Interestingly, AKAP13 expression correlates with mTORC1 activation and overall patient survival in LUAD.

AKAP13 is a 2817 amino acid protein containing multiple domains [31], and only the region (amino acids 1–2190) on AKAP13 that contains both PKA RIIα and mTORC1 binding region modulates mTORC1 activity. AKAP13 has been reported to display GEF activity for the small GTPase RhoA resulting in the activation of p38α in cardiomyocytes [27,31,41]. Another study showed that p38α promotes p38-activated kinase MK2 (also known as MAPKAPK2) activity to phosphorylate TSC2 on Ser 1210 [33]. TSC2 Ser 1210 phosphorylation results in an increase TSC2-14-3-3 binding, which may alter the function of TSC [34]. However, in our study depletion of p38α did not appear to alter the activity of mTORC1. AKAP13 has also been shown to enhance the ERK signaling cascade [35], and it was reported that TSC2 is inhibited by ERK promoting mTORC1 activation [36]. However, the Raptor Ser 791 phosphorylation status was unchanged in cells treated with ERK inhibitors. Consistently, we previously showed that PKA can phosphorylate Raptor at Ser 791 and inhibit mTORC1 in the absence of TSC [17]. This study is the first to link AKAP13 to mTORC1. Furthermore, AKAP13 GEF activity or the ERK signaling cascade does not alter Raptor Ser 791 phosphorylation.

The mRNA levels of AKAP13 are downregulated in LUAD, which is associated with a low clinical survival rate in patients. Moreover, the degree of AKAP13 expression in different LUAD cell lines correlates with mTORC1 activity. Hyperactivated mTORC1 can been seen in LUAD. For example, epidermal growth factor receptor (EGFR) mutations are seen in LUAD, and these mutations elevate EGFR resulting in mTORC1 activation [42]. Moreover, inhibition of the mTORC1 signaling pathway in LUAD has been shown to decrease cell proliferation and induce apoptosis [43]. Perhaps targeting the GPCR-Gα_s_ signaling pathway to mTORC1 or modulating the level of AKAP13 may be beneficial for patients with LUAD.

Levels of cAMP are regulated by either the rate of synthesis via adenylyl cyclase activity or the rate of degradation through a large family of cAMP-hydrolyzing phosphodiesterases (PDEs) [44]. PDEs break the phosphodiester bond and convert cAMP to 5’AMP [45]. It will be crucial to identify the PDEs that are involved in GPCR-Gα_s_ signaling pathway to mTORC1. PDEs inhibitors are currently available and are being used for a wide-range of diseases [46,47]. Targeting GPCRs in combination with a specific PDE inhibitors may significantly enhance intracellular cAMP to potently inhibit and turn off mTORC1.

## Materials and methods

### Antibodies

The following antibodies were purchased from Cell signaling and used at the indicated dilution for western blot analysis: pS6K1 (#9234, 1:1000), S6K1 (#9202, 1:1000), 4EBP1 (#9452, 1:000), p4EBP1 (#9456, 1:000), pCREB (#9198, 1:1000), CREB (#9197, 1:1000), pULK1 (#6888, 1:1000), ULK1 (#6888, 1:1000), Actin (#3700, 1:1000), p38 MAPK (#8690, 1:1000), pERK (#9101, 1:1000), ERK (#9102, 1:1000), mTOR (#2983, 1:1000), Raptor (#2280, 1:1000), mLST8 (#3227, 1:1000), Vinculin (#4650, 1:1000), pPKA Sub (RRXS*/T*) (#9624, 1:1000), PKA Cat α/β (#4782, 1:1000), LAMP2 (#49067, 1:1000) Lamin A/C (#2032, 1:1000), Tubulin (#2144, 1:1000). Flag (#F3165, 1:1000) was obtained from Sigma. HA (#sc-7392, 1:1000) was from Santa Cruz Biotechnology. AKAP8L (#GTX115831, 1:1000) was from GeneTex. AKAP13 (#A301-404A, 1:500), PKA RIα (#A303-683A, 1:1000), PKA RIIα (#A301-670A, 1:1000) was from Bethyl Laboratories. Antibodies used for the immunofluorescent microscopy experiments: mTOR (#2983, 1:200) was purchased from Cell signaling. LAMP2 (#13524, 1:200) was obtained from abcam. Secondary antibodies Alexa Fluor 488, 555 used for fluorescent microscopy were obtained from Invitrogen. LysoTracker Red DND-99 (#L7528) was obtained from Life Technologies.

### Chemicals

Forskolin (#1099) and 3-isobutyl-1-methylxanthine (IBMX, #2845) were obtained from Tocris. SCH772984 (#S7101) was obtained from Selleckchem. PD0325901 (#444968) was from Sigma.

### Cell lines and tissue culture

Most cell lines (HEK293A (including HEK293A Raptor S791A mutant cells), HCT116, DFCI032, H2126, H2887, A549 cells were maintained at 37°C with 5% CO_2_, cultured in high-glucose DMEM (#11965–092 from Invitrogen) supplemented with 10% FBS (#F2442 from Sigma), and penicillin/streptomycin (#P0781 from Sigma, 100 units penicillin and 100 μg streptomycin/mL). These cells were tested using morphology, karyotyping, and PCR-based approaches to confirm their identity—these assays include COI analysis and STR profiling. DFCI032, H2126, H2887, A549 cells were from Dr. John Minna’s lab and authenticated by DNA fingerprinting using the PowerPlex 1.2 kit (Promega). We routinely test cells for mycoplasma using Bulldog Bio Inc E-MYCO MYCOPLASMA PCR KIT 48 (#NC0767644 from Fisher).

### Immunofluorescent microscopy

Cells were seeded in 24 well plates on coverslips 1 day prior to experimentation. Coverslips were pretreated with 5 μg/mL of fibronectin (#F1141 from Sigma) at 37°C for 16 h with a quick phosphate-buffered saline (PBS) wash prior to cell seeding. The following steps were performed at room temperature: 4% paraformaldehyde (#2280 from Electron Microscopy Sciences) in PBS was used to fix the cells for 20 min, followed by washing with PBS three times for 5 min each. 0.2% triton in PBS was used to permeabilize the cells for 10 min. Cells on coverslips were blocked in 2% BSA in TBS-Tween for 1 h, followed by washing with TBS-Tween three times for 5 min each. Primary antibodies were diluted in PBS and placed on the cells for 1–3 h, followed by washing with TBS-Tween three times for 5 min each. Secondary antibodies were diluted in PBS and placed on the cells for 1 h, followed by washing with PBS three times for 5 min each, then washed with double distilled water for 5 min. Slides were mounted with prolong gold antifade reagent with DAPI (#P-36931 from Invitrogen).

For mTOR/LAMP2 co-localization experiments: HEK293A cells were transfected with either Flag-tagged AKAP13 or Flag-tagged AKAP13 Δ1387–2190. After 24 h, cells were starved of amino acids for 2h, and then stimulated with amino acids for 1 h. The cells were processed as described above. mTOR and LAMP2 co-localization was determined by quantifying the co-localization for twenty images per group obtained using a Carl Zeiss LSM 9 microscope and quantified using Image J. The microscopy images were quantified using Squassh software in Image J

For the quantification of pS6 in EGFP-AKAP13 expressing cells: PC3 and Huh7 were transfected with EGFP-tagged AKAP13 for 24 h. Images were captured with a Carl Zeiss LSM 9 microscope and analysis was performed using ImageJ. For quantification of pS6, total cell area was selected with freehand tool in ImageJ. The mean background intensity was subtracted from total raw intensity of the cell. The corrected total cell fluorescence was then measured using the following formula: CTFC = Integrated Density- (Area of selected cell X Mean fluorescence of background).

### Lysosome immunoprecipitation

MIA Paca-2 cells stably expressing Flag-tagged or HA-tagged TMEM192 were plated in 2 of 10cm plates for the following conditions: amino acid starvation (-AA) and amino acid stimulation (+AA). Cells were then starved for 2h or starved then stimulated with +AA for 2h. Cells were then collected and HA beads (Thermo Fisher Scientific, #88836) were used for immunoprecipitation overnight in CHAPS lysis buffer [39].

### RNA isolation and real-time PCR

DFCI032, H2126, H2887, and A549 cells were washed in ice cold PBS before RNA isolation and harvested using RNAeasy Plus Mini Kit (Qiagen, #74134). RNA samples (1ug) were quantitated using the NanoDrop Lite spectrophotometer (Thermo Fisher) and complementary DNA were transcribed using the iScript Reverse Transcription Supermix (Bio-Rad #1708841). Complementary DNA were then diluted 1:4 and quantified for AKAP13 (Sigma, H1_AKAP13) expression with β-actin (Sigma, H1_ACTB) as an internal control. Quantitative PCR was done using the CFX96 real-time PCR system (Bio-Rad) with iTaq Universal SYBR Green Supermix (Bio-Rad, #172–5121). RNA expression relative to internal control was calculated using the ΔΔC_T_ method.

### cDNA transfection

The protocol for cells transfected with plasmid DNA are previously described [17]. Cells were transfected with 250 ng– 3 ug HA, HA tagged raptor and mutants (S791A, S791D), FLAG, FLAG tagged AKAP8L, AKAP13 and AKAP13 truncations (1–1387, 1388–2190, 2191–2817 and 1–2190). Medium was changed 6 hours later after the transfection, and cells were harvested 24–48 hours later.

### Cell lysis and immunoprecipitation

Cells were lysis by 0.3% CHAPS as previous described [17]. For immunoprecipitations, Pierce Protein A/G (ThermoFisher Scientific, #88802), Pierce^TM^ Anti-HA (ThermoFisher Scientific, #88836) or Anti-Flag (Sigma, #A2220) beads were used for the immunoprecipitation. Immunoprecipitates were washed three times with lysis buffer. Immunoprecipitated proteins were denatured and bioled for 5 minutes, and then analyzed via Western blot analysis.

### Generation of the Raptor S791A mutant knock-in cells

Generation of the Raptor S791A mutant knock-in cells was performed as pervious described [17].

### RNA interference

Protein expression silencing was done by lentiviral shRNA. Mission shRNA (Sigma Aldrich) was co-transfected with pLKO.1-shRNA-Control or pLKO.1-shRNA-1: (CCGGCAATAAGCAACAGAT GATATTCTCGAGAATATCATCTGTTGCTTATTGTTTTTG), together with pSMD.G and psPAX-2 (Addgene) packaging vectors (4.5 μg shRNA, 3 μg psPAX-2, 1.5 μg psMD.G with 30 μL PolyJet DNA In Vitro Transfection Reagent) into a 10 cm plate to make virus. The medium was changed 6 h later. Forty-eight hours after transfection, the viral supernatant was collected, centrifuged at 3000 x g for 10 min and added to ~50% confluent control and HEK293A cells with 8 μg/mL polybrene (#AL-118 from Sigma Aldrich). Sixteen hours after transfection the medium was again changed and puromycin (#ant-pr-1 from Invitrogen) was added to a final concentration of 5 μg/mL. Under these conditions, non-infected cells died within 24 h. shRNA used for human AKAP13 from Sigma:

**TRCN0000296007:**CCGGCAATAAGCAACAGATGATATTCTCGAGAATATCATCTGTTGCTTATTGTTTTTG

**TRCN0000296006:**CCGGTGAGAATGCAGAACGTTTAAACTCGAGTTTAAACGTTCTGCATTCTCATTTTTG

**TRCN0000037971:**CCGGGCTGAAGATGATGTAGTGTTTCTCGAGAAACACTACATCATCTTCAGCTTTTTG

**TRCN0000037972:**CCGGGCAGTTCTTCTCACTGACATTCTCGAGAATGTCAGTGAGAAGAACTGCTTTTTG

#### Generation of AKAP13 knockout cells using CRISPR/Cas9 genome editing

The 20 nucleotide guide sequences targeting human AKAP13 were designed using the CRISPR design tool at https://portals.broadinstitute.org/gpp/public/[48] and cloned into the expression vector SpCas9-2A-Puro V2.0 (pX459) V2.0 (Addgene #62988). The guide sequence targeting Exon 7 of human AKAP13 are shown below:

AKAP13 5’ ATTCGAATGAGCCTGATACG 3’

The detail protocol for AKAP13 knockout are previously described [17].

### Cell size

Cells were plated in triplicate in 35 mm dishes and grown until 80% confluent. Cells were lifted up by trypsin (Sigma, #T3924) and resuspended in pre-warmed 1 x PBS. Cell number and size was measured using a Z2 Coulter Particle Count and Size Analyzer (Beckman Coulter), and processed using Z2 Accucomp software (Beckman Coulter).

### Cell proliferation

1 x 10^5^ cells were plated in triplicate in 6-well plates for proliferation. After 48 hours, cells were lifted up and mixed with Trypan Blue solution (Sigma, #T8154) at 1:1 ratio. Then, cells were placed on counting slides (Bio-Rad, #145–0011) and counted using a Bio-Rad TC20 Automated Cell Counter (Bio-Rad, #1450102).

### Colony formation

Cells (shGFP HEK293A, shAKAP13 HEK293A, DFCI032, H2126, H2887, A547) were seeded in 6 well plates at a density of 1000 cells for colony formation. After 14 days, culture medium was removed cells fixed with 4% paraformaldehyde and stained with 0.05% crystal violate.

### Mouse xenografts

A549 stable cell lines expressing control shRNA (shGFP) or shRNA targeting AKAP13 (shAKAP13) were generated. Subcutaneous xenografts were performed on NOD SCID mice (n = 7 each). Mice were injected subcutaneously in the lower flank with 2 x 10^6^ A549 cells stably expressing shGFP or shAKAP13. Tumor diameters were measured with digital calipers, and volume was calculated: Volume = (width)2 x length x 0.52.

### Lifetime imaging and fluctuation analysis of Raptor-AKAP13 interaction

Fluorescence Lifetime Imaging Microscopy (FLIM) data were collected using an Alba fluorescence correlation spectrometer (ISS, Champaign, IL), connected to a Nikon TE2000-U inverted microscope (Nikon, Melville, NY) with x-y scanning mirror and a PlanApo VC 60 x 1.2 NA water objective as previously described [49,50]. Two-photon excitation was provided by a Chameleon Ultra (Coherent, Santa Clara, CA) tuned to 900 nm for FLIM data collection to minimize the amount of direct YFP excitation. Cells were imaged using a temperature and humidity-controlled stage at 37°C with an objective wrap heater (Tokai Hit, Fujinomiya, Sizuoka, Japan) to mimic the environment of the incubator and minimize temperature drift. FLIM measurements were obtained with an ISS A320 FastFLIM box with photo multiplier detector joined to the Ti:Sapphire laser that created 80 fs pulses at a reptition rate of 80 MHz (H7422P-40, Hamamastu, Hamamastu City, Japan). A 680 nm short-pass filter (FF01-680; Semrock, Rochester, NY) and dichroic mirror (480/40x,535lpxr,550/50m Chroma, Bellows Falls, VT) was used to spectrally filter the emission of the fluorophores with separate photo multiplier tubes.

### FLIM-FRET data analysis

The phasor analysis, which was performed using SimFCS Software (E. Gratton, UCI LFD), was used to quantify FRET between EGFP-tagged AKAP13 and YFP-tagged Raptor [51–55]. Briefly, the fluorescence decay is converted into the sine and cosine components according to the equations defined in [51,52]. The pixels from the FLIM scan provide a single phasor point thus enabling spatial localization to distinct fluorescence decays. The algebraic conversion associated with the phasor analysis makes it possible to determine fractional contribution among multiple independent species [51,56]. Due to high spectral overlap between EGFP and YFP, energy transfer was calculated by measuring the phasor changes associated with the acceptor YFP. If energy transfer occurs, the YFP pixels will move outside the universal circle due to a lengthening of the acceptor lifetime [51,54]. The contribution of autofluorescence, donor bleed through, and direct acceptor excitation are taken into consideration through the rule of linear combination, with each being determined independently [51,55,56]. The combination of phasor clusters between acceptor phasor and FRET state of the samples represents the varying contributions of direct excitation and FRET acceptor fluorescence in any one pixel. By moving the phasor cursor along between the two points, we can calculate the FRET efficiency and number of pixels displaying FRET.

### Subcellular fractionation

HEK293A cells were plated in 1 x 15 cm dishes per condition and allowed to grow overnight. Cells were harvested by trypsinization after 1 hr treatment with or without 10 μM Forskolin and 200 μM IBMX treatment and spun at 3000 rpm for 5 min at 4°C. The remaining steps were performed on ice or spun at 4°C, followed by lysing according to a previously published protocol [57]. Cells were washed twice with cold 1X PBS and spun at 4000 rpm for 5 min. Cells were resuspended in 1mL of HNMEK lysis buffer (20 mM HEPES pH 7.4, 50 mM NaCl, 2 mM MgCl_2_, 2 mM EDTA, 10 mM KCl, 50 nM EGTA, protease inhibitors) and incubated for at least 20 min, then lysed using a Dounce homogenizer. Lysates were centrifuged at 750 g for 10 min to remove nuclei and cell debris. The supernatant was collected and centrifuged at 12500 g for 10 min to pellet organelle fraction (P2). Cytoplasmic (S2) fraction was collected and kept for analysis. The P2 fraction was washed twice in HNMEK lysis buffer, and centrifuged at 12500 g for 10 min. The pellet was resuspended in RIPA lysis buffer (50 mM Tris pH 8.0, 150 mM NaCl, 5 mM EDTA pH 8.0, 1% Nonidet P-40, 0.5% deoxycholate, protease inhibitors). Input (WCL), P2 and S2 fractions were collected or resuspended in equal volume.

### Statistical analysis

Statistical analyses were conducted using Prism 8 (GraphPad Software). Bar diagrams are represented as the mean of at least 3 technical replicates ± standard error of the mean (SEM). Significance was analyzed using student’s T-TEST and ANOVA.

## Supporting information

S1 Fig AKAP8L does not regulate mTORC1 activity. Elevated AKAP8L levels do not decrease mTORC1 activity. Flag-tagged AKAP8L (0–3 μg) was overexpressed in human embryonic kidney 293A (HEK293A) cells for twenty-four hours. mTORC1 activity was analyzed by protein immunoblotting for the phosphorylation status of S6K1 (pS6K1) at Thr389, 4EBP1 (p4EBP1) at Thr37 and Thr46, and ULK1 (pULK1) at Ser758. S6K, 4EBP1, ULK1, and Actin were probed as loading controls.
(TIFF)

S2 Fig mTORC1 regulation via AKAP13 is not though p38α or ERK. **(A)** The GEF activity of AKAP13 does not regulate mTORC1. Human embryonic kidney 293A (HEK293A) cells were transfected with control siRNA or siRNA targeting p38α. mTORC1 activity was analyzed by protein immunoblotting for the phosphorylation status of S6K1 (pS6K1) at Thr389, 4EBP1 (p4EBP1) at Thr37 and Thr46, and ULK1 (pULK1) at Ser758. S6K, 4EBP1, ULK1, and Actin were probed as loading controls. **(B)** AKAP13 does not regulate Raptor Ser 791 phosphorylation though the Raf-MEK-ERK signaling pathway. HA-tagged Raptor was expressed in HEK293A cells for twenty-four hours, and then treated with or without ERK inhibitors (SCH77 and PD03) for 1 h. The cells were treated with 10 μM forskolin and 200 μM IBMX for 1 h, and HA immunoprecipitates (IPs) were analyzed by immunoblotting for HA-tagged Raptor and phospho-PKA substrate antibody (pPKASub (RRXS*/T*)). Vinculin and ERK were used as loading controls.
(TIFF)

S3 Fig A Raptor Ser 791 phosphomimetic does not alter cell size and proliferation. **(A)** Expression of Raptor Ser 791 mutated to Asp 791 (S791D) in WT and AKAP13 KO HEK293A cells. Cells were probed for HA-tagged Raptor S791D. Actin was a loading control. **(B)** Expression of Raptor S791D does not alter AKAP13 regulated cell proliferation in WT or AKAP13 KO HEK293A cells. Cell proliferation in WT and AKAP13 KO HEK293A cells expressing HA or HA-tagged Raptor S791D was analyzed. P- value: WT HA vs. AKAP13 KO HA p<0.01, WT HA-tagged Raptor S791D vs. AKAP13 KO HA-tagged Raptor S791D p<0.01, WT HA vs. AKAP13 KO HA-tagged Raptor S791D p<0.001. **(C)** Expression of Raptor S791D does not impact AKAP13 regulated cell size in WT or AKAP13 KO HEK293A cells. Cell size in WT and AKAP13 KO expressing HA or HA Raptor S791D was analyzed. P-value: WT HA vs. AKAP13 KO HA p<0.01, WT HA-tagged Raptor S791D vs. AKAP13 KO HA-tagged Raptor S791D p<0.001, WT HA vs. AKAP13 KO HA-tagged Raptor S791D p<0.01. **(D)** Expression of Raptor S791D in LUAD cells (DFC1032, H2126, H2887, A549). Cells were probed for HA-tagged Raptor S791D. Actin was a loading control. **(E, F)** Overexpression of HA-tagged Raptor S791D mimetic does not impact cell proliferation and cell size in DFCI032 cells. **(G, H)** Overexpression of Raptor S791D mimetic does not impact cell proliferation and cell size in H2126 cells. **(I, J)** Overexpression of Raptor S791D mimetic does not impact cell proliferation and cell size in H2887 cells. **(K, L)** Overexpression of Raptor S791D mimetic does not impact cell proliferation and cell size in A549 cells.
(TIFF)

S4 Fig Expression of AKAP13 alters mTORC1 activity in other cancer cell lines. **(A)** Prostate (PC3) and **(B)** liver (Huh7) cancer cells were transfected with EGFP-tagged AKAP13. *Top-* Cells with high EGFP-tagged AKAP13 do not have high mTORC1 activity. Represented image of cells with EGFP-tagged AKAP13 and S6 phosphorylation (pS6). Scale bar = 5μM. *Bottom-* The relative intensity of S6 phosphorylation (pS6, mTORC1 activity) was measured. P-value: PC3 cells with no EGFP-tagged AKAP13 (-EGFP AKAP13) vs cells with EGFP-tagged AKAP13 (+EGFP AKAP13) p<0.001, Huh7 cells with no EGFP-tagged AKAP13 (-EGFP AKAP13) vs cells with EGFP-tagged AKAP13 (+EGFP AKAP13) p<0.0001.
(TIFF)

S1 Data Excel files containing all numerical data underlying the graphs herein presented. (XLSX)
