# Supplementary figures and images for: AKAP13 couples GPCR signaling to mTORC1 inhibition
Source: PLoS Genet. 2021 Oct 21;17(10):e1009832. doi: 10.1371/journal.pgen.1009832 (PMC8570464; doi:10.1371/journal.pgen.1009832)

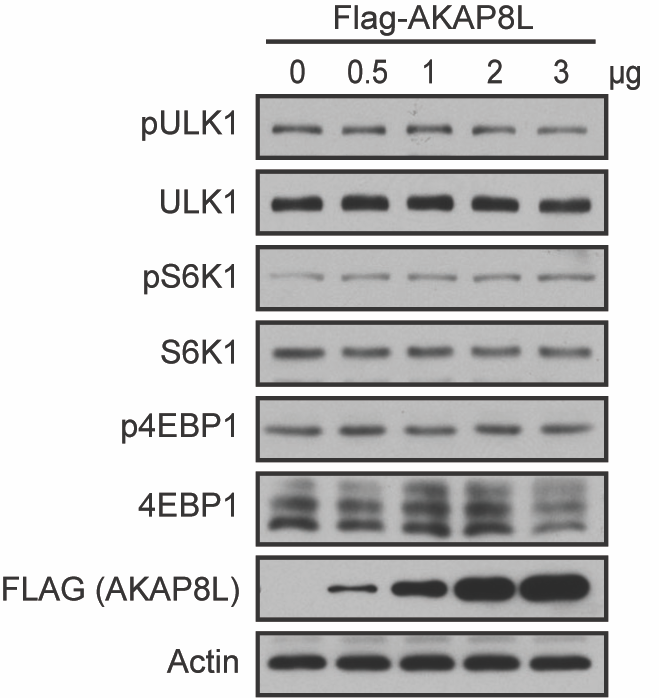

Supplement: S1 Fig — Elevated AKAP8L levels do not decrease mTORC1 activity. Flag-tagged AKAP8L (0–3 μg) was overexpressed in human embryonic kidney 293A (HEK293A) cells for twenty-four hours. mTORC1 activity was analyzed by protein immunoblotting for the phosphorylation status of S6K1 (pS6K1) at Thr389, 4EBP1 (p4EBP1) at Thr37 and Thr46, and ULK1 (pULK1) at Ser758. S6K, 4EBP1, ULK1, and Actin were probed as loading controls. (TIFF) [file pgen.1009832.s001.tiff]

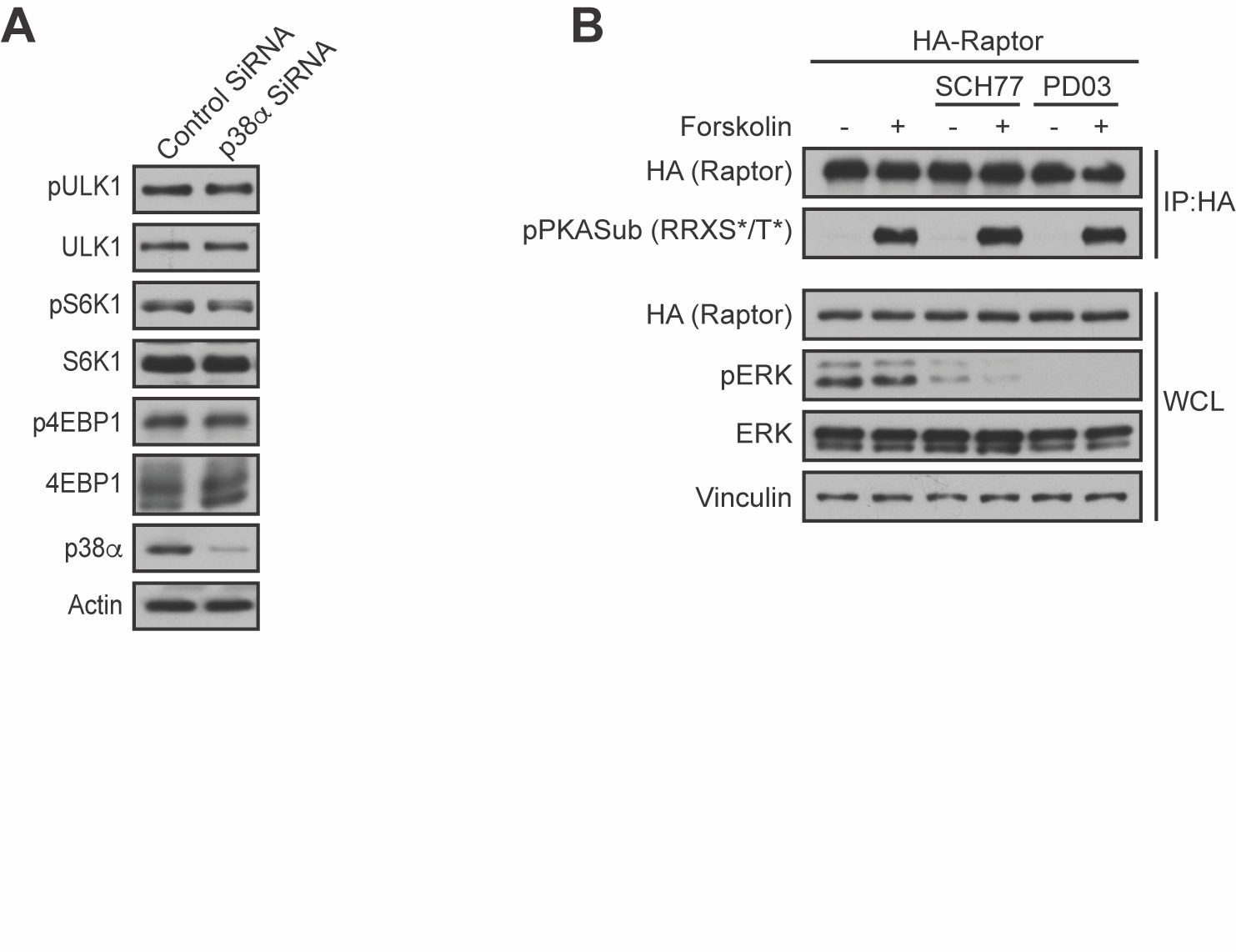

Supplement: S2 Fig — (A) The GEF activity of AKAP13 does not regulate mTORC1. Human embryonic kidney 293A (HEK293A) cells were transfected with control siRNA or siRNA targeting p38α. mTORC1 activity was analyzed by protein immunoblotting for the phosphorylation status of S6K1 (pS6K1) at Thr389, 4EBP1 (p4EBP1) at Thr37 and Thr46, and ULK1 (pULK1) at Ser758. S6K, 4EBP1, ULK1, and Actin were probed as loading controls. (B) AKAP13 does not regulate Raptor Ser 791 phosphorylation though the Raf-MEK-ERK signaling pathway. HA-tagged Raptor was expressed in HEK293A cells for twenty-four hours, and then treated with or without ERK inhibitors (SCH77 and PD03) for 1 h. The cells were treated with 10 μM forskolin and 200 μM IBMX for 1 h, and HA immunoprecipitates (IPs) were analyzed by immunoblotting for HA-tagged Raptor and phospho-PKA substrate antibody (pPKASub (RRXS*/T*)). Vinculin and ERK were used as loading controls. (TIFF) [file pgen.1009832.s002.tiff]

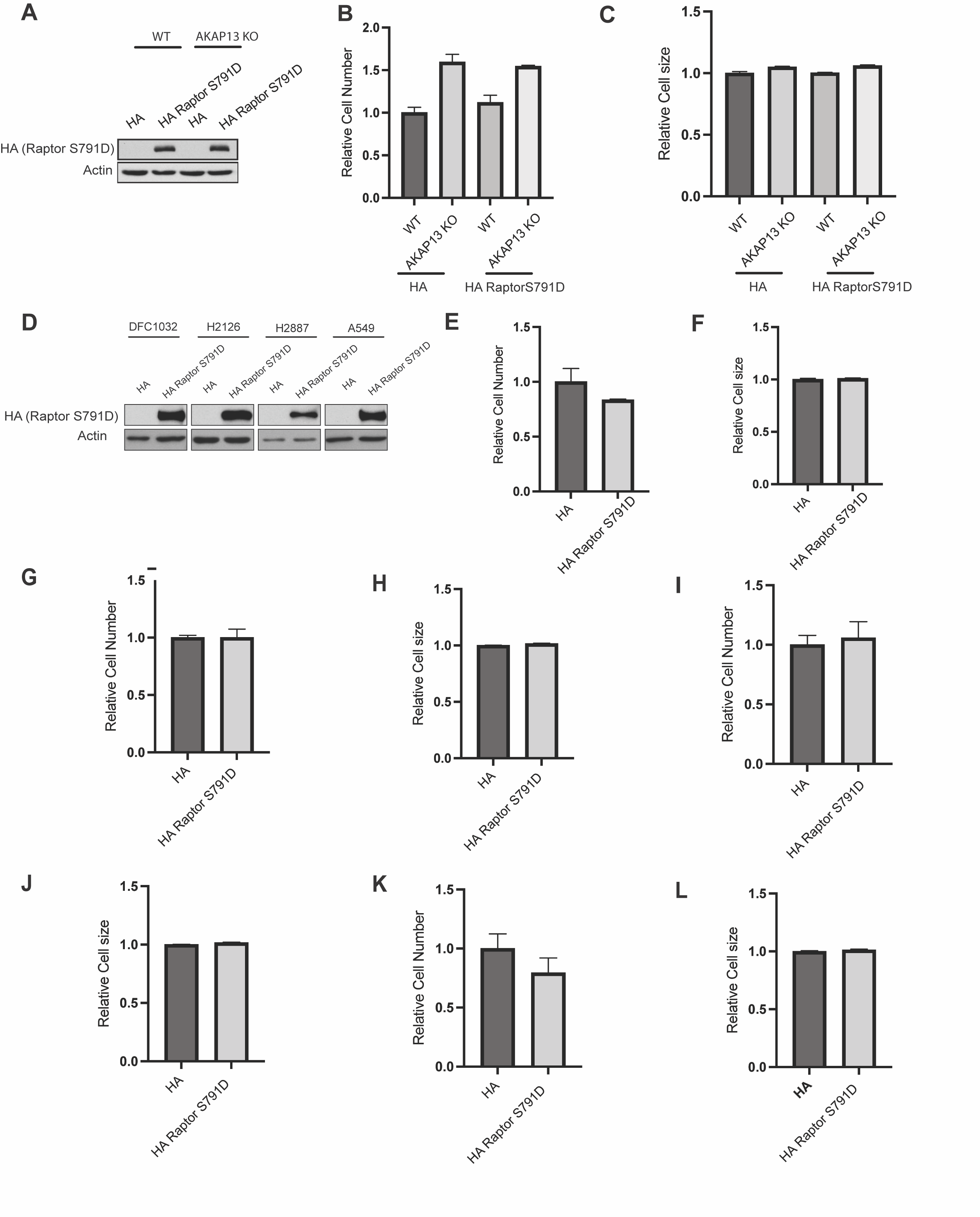

Supplement: S3 Fig — (A) Expression of Raptor Ser 791 mutated to Asp 791 (S791D) in WT and AKAP13 KO HEK293A cells. Cells were probed for HA-tagged Raptor S791D. Actin was a loading control. (B) Expression of Raptor S791D does not alter AKAP13 regulated cell proliferation in WT or AKAP13 KO HEK293A cells. Cell proliferation in WT and AKAP13 KO HEK293A cells expressing HA or HA-tagged Raptor S791D was analyzed. P- value: WT HA vs. AKAP13 KO HA p<0.01, WT HA-tagged Raptor S791D vs. AKAP13 KO HA-tagged Raptor S791D p<0.01, WT HA vs. AKAP13 KO HA-tagged Raptor S791D p<0.001. (C) Expression of Raptor S791D does not impact AKAP13 regulated cell size in WT or AKAP13 KO HEK293A cells. Cell size in WT and AKAP13 KO expressing HA or HA Raptor S791D was analyzed. P-value: WT HA vs. AKAP13 KO HA p<0.01, WT HA-tagged Raptor S791D vs. AKAP13 KO HA-tagged Raptor S791D p<0.001, WT HA vs. AKAP13 KO HA-tagged Raptor S791D p<0.01. (D) Expression of Raptor S791D in LUAD cells (DFC1032, H2126, H2887, A549). Cells were probed for HA-tagged Raptor S791D. Actin was a loading control. (E, F) Overexpression of HA-tagged Raptor S791D mimetic does not impact cell proliferation and cell size in DFCI032 cells. (G, H) Overexpression of Raptor S791D mimetic does not impact cell proliferation and cell size in H2126 cells. (I, J) Overexpression of Raptor S791D mimetic does not impact cell proliferation and cell size in H2887 cells. (K, L) Overexpression of Raptor S791D mimetic does not impact cell proliferation and cell size in A549 cells. (TIFF) [file pgen.1009832.s003.tiff]

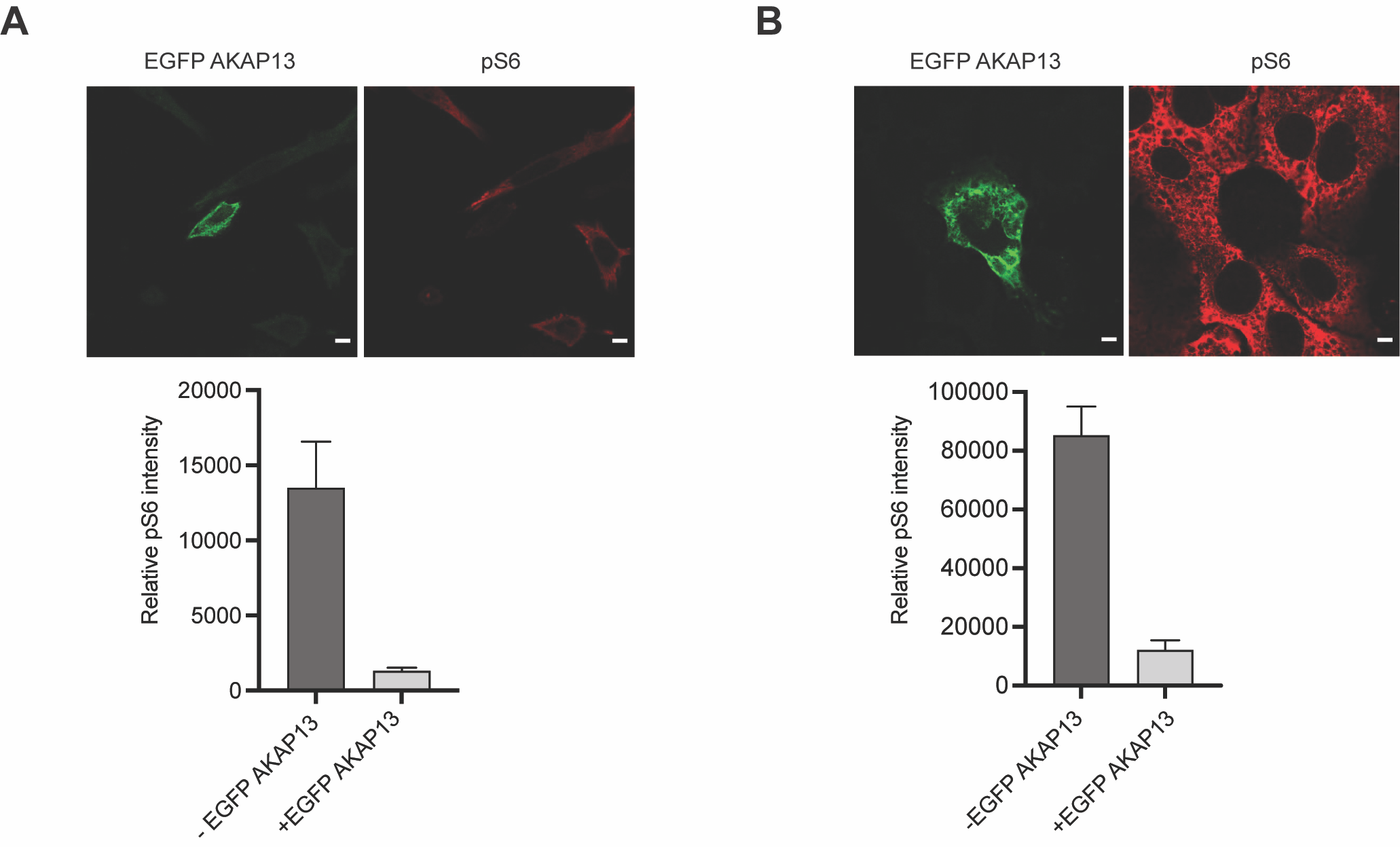

Supplement: S4 Fig — (A) Prostate (PC3) and (B) liver (Huh7) cancer cells were transfected with EGFP-tagged AKAP13. Top- Cells with high EGFP-tagged AKAP13 do not have high mTORC1 activity. Represented image of cells with EGFP-tagged AKAP13 and S6 phosphorylation (pS6). Scale bar = 5μM. Bottom- The relative intensity of S6 phosphorylation (pS6, mTORC1 activity) was measured. P-value: PC3 cells with no EGFP-tagged AKAP13 (-EGFP AKAP13) vs cells with EGFP-tagged AKAP13 (+EGFP AKAP13) p<0.001, Huh7 cells with no EGFP-tagged AKAP13 (-EGFP AKAP13) vs cells with EGFP-tagged AKAP13 (+EGFP AKAP13) p<0.0001. (TIFF) [file pgen.1009832.s004.tiff]
